# Integrated Whole-Transcriptome Profiling and Bioinformatics Analysis of the Polypharmacological Effects of Ganoderic Acid Me in Colorectal Cancer Treatment

**DOI:** 10.3389/fonc.2022.833375

**Published:** 2022-04-27

**Authors:** Nianhong Chen, Guoqing Wan, Xiaobin Zeng

**Affiliations:** ^1^Center Lab of Longhua Branch and Department of Infectious Disease, Shenzhen People’s Hospital, The Second Clinical Medical College, Jinan University; The First Affiliated Hospital, Southern University of Science and Technology, Shenzhen,China; ^2^Guangdong Provincial Key Laboratory of Regional Immunity and Diseases, Medicine School of Shenzhen University, Shenzhen, China; ^3^Laboratory of Signal Transduction, Department of Radiation Oncology, Memorial Sloan-Kettering Cancer Center, New York, NY, United States

**Keywords:** ganoderic acid Me, colorectal cancer, whole-transcriptomic analysis, network analysis, molecular docking study, protein–protein interaction network

## Abstract

Ganoderic acid Me (GA-Me) is a natural bioactive compound derived from *Ganoderma lucidum*. Our present results suggested that GA-Me inhibited proliferation, induced DNA fragmentation and significantly activated caspase-9 and caspase-3 in HCT116 cells. As shown in our previous studies, GA-Me targets several genes to prevent cancer, including colorectal cancer (CRC). Thus, we hypothesized that GA-Me might be a multitarget ligand against cancer. However, its exact mechanism in CRC remains unclear. Here, whole-transcriptome sequencing was employed to assess the long noncoding RNA (lncRNA), circular RNA (circRNA), microRNA (miRNA), and messenger RNA (mRNA) profiles of GA-Me-treated HCT116 cells. In total, 1572 differentially expressed (DE) lncRNAs, 123 DEcircRNAs, 87 DEmiRNAs, and 1508 DEmRNAs were identified. DCBLD2 and RAPGEF5 were validated as two core mRNAs in the DElncRNA, DEcircRNA, and DEmiRNA networks. Gene Ontology (GO) and Kyoto Encyclopedia of Genes and Genomes (KEGG) analyses revealed the biological functions and potential mechanisms of TCONS-00008997, XR-925056.2, circRNA-07908, hsa-miR-100-3p, hsa-miR-1257, hsa-miR-3182, NAV3, ADAM20, and STARD4, which were altered after GA-Me treatment. The regulatory relationships of the XR-925056.2-hsa-miR-3182-NAV3/ADAM20/STARD4, circRNA-07908|Chr22:38986298-39025349-hsa-miR-3182-NAV3/ADAM20, ENST00000414039/ENST00000419190-novel874_mature-MMP9 and circRNA-00314|Chr1:35470863-35479212/circRNA-05460|Chr17:72592203-72649268-novel874_mature-MMP9 immune-regulatory networks involved both noncoding RNAs (ncRNAs) and mRNAs. Molecular docking studies showed that Zn^2+^ and the His201, His205, His211, Glu202, and Ala165 residues of MMP2 contributed to its high affinity for GA-Me. Zn^2+^ and the Glu402 and Gly186 residues of MMP9 are important for its interaction with GA-Me. Our results suggested and confirmed that GA-Me is a potential multitarget lead compound for CRC treatment with unique polypharmacological advantages.

## Introduction

Colorectal cancer (CRC) is a common, often fatal malignancy and the third leading cause of cancer in both men and women (1). According to global statistics, CRC affected more than 1.8 million new patients and was expected to cause 0.88 million cancer-related deaths in 2018 (2). In the United States, the number of new CRC cases reached 104,610, and the number of deaths reached 53,200 in 2020 (3). In recent years, because of the high recurrence rate and poor prognosis, the current therapies for CRC do not provide a very effective therapeutic outcome, and its mortality rate is still unacceptably high, even with advances in CRC diagnosis and therapies (4). Therefore, new CRC therapies and bioactive compounds are urgently being researched and developed.

Noncoding RNAs (ncRNAs), which are abundant in cells, regulate the expression of functional proteins but do not encode proteins. Based on their size and biogenesis pathways, ncRNAs are usually subdivided into several families, including long ncRNAs (lncRNAs, with a length >200 nucleotides), circular RNAs (circRNAs, with a closed continuous loop), and microRNAs (miRNAs, with <200 nucleotides), and some RNA–protein complexes regulate gene expression (5). Based on accumulating evidence, ncRNAs are involved in important biological functions (6). Moreover, multiple studies have indicated that ncRNA mutations and dysregulation are involved in various human diseases, especially cancers (7). Colorectal cancer is characterized by genetic and epigenetic modifications, and ncRNAs are emerging as important regulators of gene expression in CRC (8, 9).

Approximately 60% of current antitumor drugs are derived from natural products, and natural compounds are recognized as an invaluable resource in drug discovery and have been developed for treating various diseases, including cancer (10). As a natural compound that has great potential as a resource, *Ganoderma lucidum* (Fr.) Karst (Polyporaceae), a traditional Chinese medicinal mushroom, has been used for centuries in East Asia to prevent and treat various human diseases, including cancer (11–17). Triterpenes from *Ganoderma lucidum* induce cell cycle arrest and apoptosis and inhibit proliferation, angiogenesis, invasion and metastasis in human and murine carcinoma cells and mice (14, 18–23). As described in our previous study, our group purified ganoderic acid Me (GA-Me), a natural bioactive compound from *G. lucidum*, and showed that it induced HCT116 cell apoptosis by upregulating p53, Bax and caspase-3 (16, 17), suppressed tumor invasion by inhibiting MMP2 and MMP9 expression (11), and inhibited tumor growth and lung metastasis by increasing interleukin-2 (IL-2)/interferon-γ (IFN-γ) expression and activating immune natural killer (NK) cells (12). GA-Me also induced T cell apoptosis, reduced the number of CD8^+^ T cells, and increased Treg-mediated immunosuppression by affecting the indoleamine 2,3-dioxygenase (IDO)-mediated microenvironment in lung tumors (24). Additionally, GA-Me obviously reversed multidrug resistance by inhibiting the expression of MDR1, MRP1 and MRP2 (25). These results implied that GA-Me may target several proteins individually or simultaneously.

Because drug development has recently tended toward systems-level polypharmacology, a more systems biology-oriented approach considering pleiotropy in biological networks at a molecular and cellular level (26), this approach is able to identify the ligands that bind several selected therapeutic targets. Small-molecule compounds may be able to interact with diverse targets individually or simultaneously (27). Our previous studies confirmed the antitumor efficacy of GA-Me, but the systematic mechanisms of its anti-CRC effects remain largely unknown. In addition, its system-level polypharmacological profile still must be elucidated to clarify its potential as an antitumor lead compound for drug development. Therefore, we hypothesized that GA-Me might be a multitarget ligand, but its polypharmacological effects are still largely unknown and should be elucidated with a systems biology approach.

Hence, large-scale systematic data are required for the deep interpretation of the mechanisms underlying the anti-CRC effects of GA-Me. However, few studies have been published describing the effects of GA-Me on CRC at the transcriptomic level, and many questions remain unanswered regarding the integration of ncRNA and mRNA expression profiling approaches with respect to GA-Me treatment. Thus, whole-transcriptome and miRNA sequencing were applied to profile the coding transcriptome and ncRNA changes after GA-Me treatment of HCT116 cells. Then, the identified differentially expressed (DE) mRNAs and DEncRNAs were explored to determine their biological functions and signaling pathways. In particular, this study reports a systems-level polypharmacological profile based on Gene Ontology (GO) and Kyoto Encyclopedia of Genes and Genomes (KEGG) analyses and molecular docking studies *in silico*. Additionally, coexpression networks were constructed to disclose the multitarget interactions according to these sequencing results and the bioinformatics analysis. Furthermore, molecular docking studies showed that Zn^2+^ and the His201, His205, His211, Glu202, and Ala165 residues of MMP2 contributed to its high affinity for GA-Me. Zn^2+^ and the Glu402 and Gly186 residues of MMP9 are important for its interaction with GA-Me. Using the Search Tool for the Retrieval of Interacting Genes/Proteins (STRING, https://string-db.org/), the protein symbols corresponding to the top protein-coding genes were annotated, and their interactions were visualized by constructing a PPI network. Our results might provide novel insights into the polypharmacological profile of GA-Me and its anti-CRC efficacy to improve the application of GA-Me lead compounds.

## Materials and Methods

### Materials

McCoy’s 5A modified medium and dimethyl sulfoxide (DMSO) were obtained from Sigma-Aldrich (St. Louis, MO, USA). PrimeScript™ reverse transcriptase was obtained from TransGen Biotech, Beijing, China. The generated transcripts were then subjected to qRT–PCR using the SYBR Green PCR Reagent Kit, and SYBR Green Master mix was obtained from Takara (Tokyo, Japan).

GA-Me was purified with semipreparative liquid chromatography and identified using ESI-MS, ^13^C NMR, and ^1^HNMR methods (28), and the purity was approximately 99% (11, 16, 17). A stock solution of GA-Me was prepared in DMSO and stored at -80°C. Further dilutions were prepared with McCoy’s 5A modified medium just before use. The final concentration of DMSO was less than 0.1%.

### Cell Culture

The human highly metastatic lung cancer cell line 95-D, an epithelial-like, non-small cell lung epithelial cancer cell line, was cultured in RPMI 1640 medium. HCT116 colon carcinoma cells were cultured in McCoy’s 5A modified medium and 10% (v/v) dialyzed heat-inactivated FBS at 37°C in a humidified atmosphere composed of 95% air and 5% CO_2_ (17, 29).

### Cell Proliferation Assay

For the proliferation assay, cells were grown at a density of 1×10^6^ cells/ml in 6-well Costar plates containing McCoy’s 5A Modified Medium at 37°C for 4 days in a 95% air, 5% CO_2_ incubator. Aliquots of cells and medium were removed at 2-day intervals. Cultures containing 18.1 µM GA-Me were examined, and viable cell numbers were counted at each time interval with a trypan blue staining cell viability assay after the cells were harvested by trypsinization (30).

### DNA Fragmentation Assay

DNA fragmentation in apoptotic cells was determined using gel electrophoresis. The cells (2×10^5^) were treated with 83.4 µM Dox (doxorubicin) and the indicated concentrations of GA-Me for 24 h, then washed with PBS, followed by an incubation with extraction buffer (10 mM Tris, pH 8.0; 0.1 mM EDTA, 0.5% SDS; and 20 μM RNase) at 37°C for 1 h. Subsequently, 100 μg/ml of proteinase K was added, and the sample was incubated at 50°C for 3 h. DNA was extracted with phenol/chloroform and chloroform. The aqueous phase was precipitated with two volumes of 100% ethanol and 1/10 volume of 3 M sodium acetate for 30 min on ice. The DNA pellet was then washed with 70% ethanol and suspended in 50 μl of Tris-EDTA buffer. The absorbance of the DNA solution at 260 and 280 nm was determined using a spectrophotometer. The extracted DNA (40 μg/lane) was subjected to electrophoresis on 2% agarose gels. The gels were stained with ethidium bromide and then photographed (30, 31).

### Caspase Activity Assay

Caspase 3 and 9 catalytic activities were assessed using Caspase-Glo 3/7 and 9 assay kits (Promega, USA) according to the manufacturers’ instructions (16, 32). For Caspase-Glo 9 substrates, the mixture was treated with the inhibitor MG132. Briefly, HCT116 cells were seeded at a density of 2×10^3^ cells/well in a white-walled 96-well plate and cultured for 8 h. HCT116 cells were subsequently incubated with or without 54.3 μM GA-Me for 24 hours. Then, 100 μl of medium were carefully removed from each well without disturbing the cells before 100 μl of Caspase-Glo reagent were added to the cells. The plate was placed on an orbital shaker for 30 s and subsequently incubated at room temperature for 1 hour. The luminescence was measured using a GENios Pro plate reader (Tecan, Research Triangle Park, NC, USA).

### RNA Extraction and Quality Monitoring

The transcriptomic analysis was performed on untreated HCT116 cells and HCT116 cells treated with 54.3 μM GA-Me for 24 h. First, total RNA was extracted from HCT116 cells analyzed in three independent experiments using TRIzol reagent and treated with DNase I (Invitrogen, Carlsbad, CA, USA) according to the manufacturer’s protocol. RNA quantity and quality were measured using a NanoDrop 2000 spectrophotometer (Thermo Fisher Scientific, Wilmington, DE, USA) and an Agilent 2100 Bioanalyzer (Agilent Technologies) to obtain an RNA integrity number (RIN) > 9.

### cDNA Synthesis and qPCR

Total RNA was extracted using TRIzol reagent (Takara, Japan). DNA was removed from the samples using DNase I (Takara, Shiga, Japan). The purity of the isolated RNA was determined, and the A260/A280 ratio of each RNA sample was greater than 1.8. The cDNA templates were synthesized using PrimeScript™ reverse transcriptase (TransGen Biotech, Beijing, China). The generated transcripts were then subjected to qRT–PCR using the SYBR Green PCR Reagent Kit (Takara, Tokyo, Japan). Quantitative assays were performed in triplicate using 1 mL of cDNA templates (1:10 dilution) and SYBR Green Master Mix (Takara, Tokyo, Japan). Data were collected using the Eppendorf MasterCycler^®^ ep RealPlex4 (Wesseling-Berzdorf, Germany). Target gene expression was normalized to that of β-actin to determine relative abundance using the 2^− CT^ method or fold change (FC) compared to the mock control using the 2^− CT^ method. The primers used in these experiments are shown in **Table 1**. The PCR protocol used included a 1 min denaturation step at 95°C followed by 40 cycles of 15 s at 95°C and 1 min at 60°C, as described in our previous studies (33). The primers are shown in **Table 1**.

**Table 1:** Primers designed for qRT–PCR validation of DEGs.

Gene Name	Direction	Sequence (5’-3’)
**USP3**	USP3-F	5’-TAGCCCAGAGTCCTTATTTTATGTT-3’
	USP3-R	5’-CGTTGAAACCGCCCTGAA-3’
**Bcl2**	Bcl2-F	5’-TTTGAGTTCGGTGGGGTCAT-3’
	Bcl2-R	5’-GAGACAGCCAGGAGAAATCAAAC-3’
**Bax**	BaxF	5’- TTCTGACGGCAACTTCAACTG-3’
	Bax-R	5’- AGGGACATCAGTCGCTTCAGT-3’
**Caspase 8**	Caspase 8-F	5’-GGGGTAATGACAATCTCGGACT-3’
	Caspase 8-R	5’-TCAAAGGTCGTGGTCAAAGC-3’
**Cyclin E1**	Cyclin E1-F	5’-AGCGGTAAGAAGCAGAGCAG-3’
	Cyclin E1-R	5’-CGCTGCAACAGACAGAAGAG-3’
**CDK6**	CDK6-F	5’-GCCCACTGAAACCATAAAGGA-3’
	CDK6-R	5’-AGGTTAGAGCCATCTGGAAACTAT-3’
**MMP9**	MMP9-F	5’-TCGTGGTTCCAACTCGGTTT-3’
	MMP9-R	5’-GCGGCCCTCGAAGATGA-3’
**IFNAR1**	IFNAR1-F	5’-GCAAAGCTCAGATTGGTCCTC-3’
	IFNAR1-R	5’-AAACCATCCAAAGCCCACA-3’

DEGs, denote differentially expressed genes.

### RNA library construction, quality control, sequencing, and transcript assembly

A small RNA library (sRNA library) was constructed with the TrueSeq Small RNA Library Prep Kit (Cat. No. RS-200-0012, Illumina San Diego CA, USA) according to the manufacturer’s instructions, and 1 μg of RNA per sample was used as the initial amount. T4 RNA ligase 1 was ligated to the 3’ end of the RNA, followed by the ligation of T4 RNA ligase 2 (truncated) to the 5’ adaptor. Afterward, the RNA was reverse transcribed to synthesize cDNAs with SuperScript II Reverse Transcriptase. Finally, small RNA libraries were obtained by screening the recovered fragments using gel separation technology. During the construction of the lncRNA library (chain-specific library for removal of ribosomal RNA), the epicenter Ribo-Zero Gold kit was used to remove ribosomal RNA. Then, rRNA-depleted RNA was fragmented and used as a template to construct the cDNA library. The qualities of the libraries were further tested using the following steps: 1) initial quantification was performed using Qubit 2.0, and the insert size of the library was tested by Agilent 2100 and 2) the qPCR method was used to accurately quantify the effective concentration of the library (effective library concentration > 2 nM). After the libraries passed quality testing, different libraries were pooled according to the amount of target data and then sequenced with the Illumina HiSeq2000 platform. Duplicate/triple samples of cells treated with the mock control or GA-Me for 24 h were used to prepare RNA libraries for sequencing with the Illumina HiSeq2000 platform (Illumina) to generate 100-bp paired-end sequencing reads. Raw data were filtered to remove adaptor sequences and low-quality reads. The remaining rRNA reads were removed by mapping to known human rRNA sequences. The clean, high-quality data were mapped to the human reference genome (GRCh37.p10/hg19) using TopHat (2.0.10). The mapped reads for each sample were independently assembled into annotated and novel transcripts using the Cufflinks (2.1.1) suite of programs. Gene expression levels were evaluated as the sum of the fragments per kilobase of exon model per million reads mapped (FPKM) in each exon. For more accurate results, samples showing estimated FPKM values less than 1.0 in either cell line were filtered (for reference, the lowest level of expression commonly used is 0.05 FPKM).

### Bioinformatics Analysis

Read counts for each RNA were generated using HTSeq (0.6.0) for each sample and analyzed using the DESeq2 (1.2.10) program to assess differential expression between control and GA-Me-treated samples (33). Significance was calculated using the Wald test, and a Benjamini–Hochberg false discovery rate cutoff of 5% was used to assess statistically significant differential expression. The lowest quartile of RNAs based on expression was excluded from further analysis. RefSeq genomic feature distribution information for coding exons, introns, 3′ and 5′ untranslated regions (UTRs), promoters (-1 kb), and transcription termination sites (+1 kb) was downloaded from the University of California, Santa Cruz (UCSC) Genome Browser (http://genome.ucsc.edu/) and analyzed using the BEDtools program. The Database for Annotation, Visualization, and Integrated Discovery (DAVID) (6.8), an online functional annotation tool, was used to conduct the gene enrichment analysis. A list of gene symbols corresponding to DERNAs was mapped to DAVID gene IDs to determine which GO biological processes (BPs) and KEGG pathways were enriched. Clustering analysis of enriched GO BPs was performed using the DAVID heuristic fuzzy multiple-linkage partitioning method. An enrichment score cutoff of 3 was used to determine significance. The base-by-base PhastCons conservation score across 100 vertebrates was downloaded from the UCSC Genome Browser. For each RNA, a mean score was calculated if a score was available for at least 80% of the bases in the sequence. PhyloCSF software was locally installed and used to determine the coding potential of the longest start-to-stop open reading frame in each RNA. The multiple species alignments needed for this analysis were prepared with the Galaxy web platform at usegalaxy.org. The overlapping host factors identified by transcriptomics methods were identified and visualized using Venny v2.0, a Venn diagram web resource (http://bioinfogp.cnb.csic.es/tools/venny/).

### GO and KEGG Pathway Analyses

We performed GO enrichment and KEGG pathway analyses with the predicted target genes of DElncRNAs, the source genes of DEcircRNAs, the predicted target genes of DEmiRNAs, and the DEmRNAs to better understand the biological functions and potential mechanisms of ncRNAs and mRNAs in the effects of GA-Me on CRC. Briefly, GO (www.geneontology.org) results consist of three components: BP, cellular component (CC), and molecular function (MF). KEGG analyses were conducted to investigate the potential significant pathways (http://www.genome.jp/kegg/).

### Construction of a Coexpression Network

The lncRNAs, circRNAs, and mRNAs that competitively bind miRNAs and serve as miRNA sponges are named competing endogenous RNAs (ceRNAs). The lncRNA-miRNA-mRNA and circRNA-miRNA-mRNA networks were constructed with Cystoscope software v3.8.0 (San Diego, CA, USA) to investigate the role and interactions between ncRNAs and mRNAs after GA-Me treatment. The lncRNA-miRNA–mRNA network analysis included two scenarios: (1) upregulated lncRNA-downregulated miRNA-upregulated mRNA and (2) downregulated lncRNA-upregulated miRNA-downregulated mRNA. Analogously, the circRNA-miRNA–mRNA pair network also covered two scenarios: (1) upregulated circRNA-downregulated miRNA-upregulated mRNA and (2) downregulated circRNA-upregulated miRNA-downregulated mRNA. Cytoscape software was used to build and visually display the networks. Different shapes and colors represent different RNA types and regulatory relationships, respectively.

### Molecular Docking Studies

We implemented SYBYL-X 1.3 in a molecular docking study to explore the detailed interactions between GA-Me and MMP2 and MMP9. The crystal structure of MMP2 (Protein Data Bank [PDB] entry: 1QIB) was downloaded from the PDB (34, 35). The MOLCAD module in SYBYL-X 1.3 was used to define the binding pocket of MMP2. Our predicted pocket was similar to the reported S1’ pocket of matrix metalloproteinases (MMPs) (36). Based on the pocket information, detailed interactions between GA-Me and MMP2 were explored with the docking program Surflex-Dock GeomX (SFXC) in SYBYL-X 1.3. The cocrystal structure of the MMP9 complex with a reverse hydroxamate inhibitor (PDB entry: 1GKC) was acquired from the PDB (37). The original ligand, STN-BUM, was used to predict the binding pocket. Afterward, it was removed in PyMOL to avoid unnecessary blocks and interactions (38). The docking results showed several hydrogen bonds, hydrophobic contacts, and conjugation to Zn^2+^-stabilized GA-Me within a defined pocket.

### PPI Network and Module Analysis

All 1508 DEmRNAs were imported into the STRING database for the PPI network analysis (39). Interactions with a composite score > 0.4 were considered statistically significant. The node proteins obtained from the STRING database were then imported into the CytoHubba plug-in of Cytoscape software to analyze and identify the top 20 crucial node proteins that were imported to the STRING database to construct the PPI network. We also analyzed the top 20 differentially expressed genes (DEGs) involved in the immune response after GA-Me treatment.

### Statistical Analysis

All data are presented as the means ± SD of triplicates. Statistical analyses were performed using Student’s *t test* (between two groups), one-way or two-way ANOVA followed by Dunnett’s multiple comparison tests (OriginPro 8 Origin Lab Inc., between three and more than three groups) to evaluate the significance of differences. For sequencing data, we analyzed DEncRNAs and DEmRNAs using DESeq (Anders and Huber, 2010) software, with *p* < 0.05 and |log2FC| > 1 as the screening criteria. The statistical significance of differences between untreated and GA-Me-treated cells was assayed, and differences with *p* < 0.05 were considered statistically significant.

## Results

### Transcriptomic Analysis of DEncRNAs and DEmRNAs in GA-Me-Treated HCT116 Cells

The cDNA and sRNA libraries of HCT116 cell samples treated with or without GA-Me were sequenced. The counts of clean reads and the mapped ratios of sequencing data are shown in **Table 2**. Overall, we detected 51392 lncRNAs (including 973 new lncRNAs), 12842 circRNAs, 2879 miRNAs (including 1375 new miRNAs), and 20030 mRNAs in our project. The top 30 downregulated and top 30 upregulated lncRNAs, circRNAs, miRNAs, and mRNAs in the GA-Me-treated cells compared with the untreated cells are listed in **Tables S1****–****S4**.

**Table 2:** Deep RNA sequencing analysis of different groups.

Sample ID		lncRNA + mRNA+ circRNA sequencing			miRNA sequencing		
		Clean reads	Q30 (%)	Mapped ratio	Clean reads	Q20 (%)	Mapped ratio
**GA-Me**	GA-Me 1	94532654	94.6	85.11%	32036838	94.51	84.36%
	GA-Me 2	109358418	94.42	85.74%	27108639	93.86	86.72%
	GA-Me 3	108789698	94.19	85.91%	27621360	93.66	85.29%
**Control**	Control 1	103013400	94.41	88.57%	29786975	94.44	83.19%
	Control 2	116198446	94.62	87.75%	29944864	93.53	83.00%
	Control 3	113590758	94.4	89.02%	30854058	95.38	83.05%

GA-Me 1, GA-Me 2, and GA-Me 3 represent three groups of HCT116 cells treated with GA-Me. Control 1, Control 2, and Control 3 represent three groups of untreated HCT116 cells.

**Figure 1:**
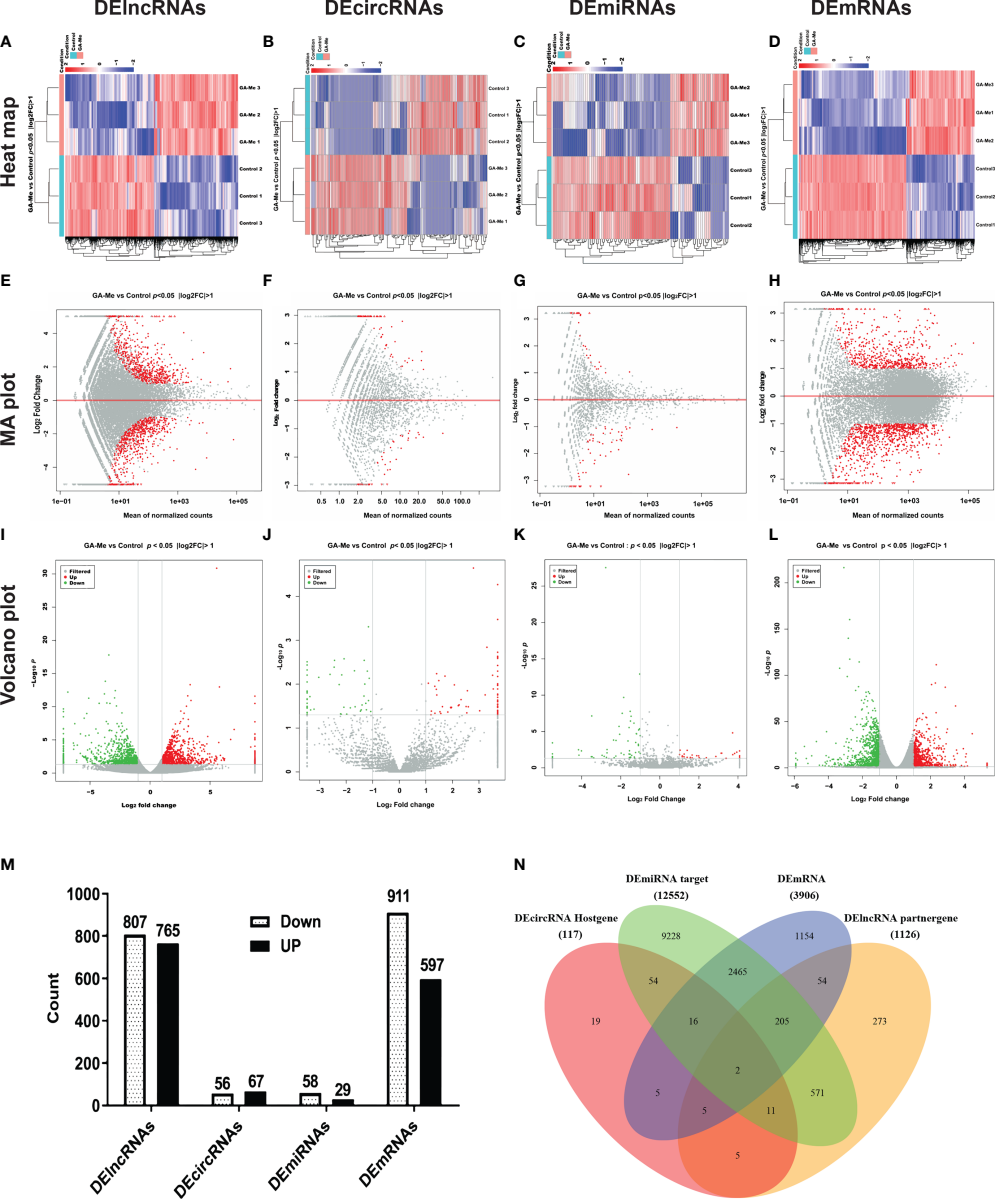
RNA-Seq revealed distinct expression levels and patterns of lncRNAs, circRNAs, miRNAs, and mRNAs in untreated and GA-Me-treated HCT116 cells. **(A–D)** Unsupervised clustering analysis comparing the expression profiles of DElncRNAs, DEcircRNAs, DEmiRNAs, and DEmRNAs between untreated and GA-Me-treated HCT116 cells. **(E–H)** MA plot of DElncRNA, DEcircRNA, DEmiRNA, and DEmRNA expression profiles between untreated and GA-Me-treated HCT116 cells. **(I–L)** Volcano plot of DElncRNA, DEcircRNA, DEmiRNA, and DEmRNA expression profiles between untreated and GA-Me-treated HCT116 cells. Histogram showing the number of up- and downregulated ncRNAs and mRNAs **(M)**. Venn diagram showing the number of overlapping DEmRNAs, DElncRNA-target trans mRNAs, DEmiRNA-target mRNAs, and DEcircRNA-host genes. RNA-Seq revealed distinct expression patterns of lncRNAs, circRNAs, miRNAs, and mRNAs between untreated and GA-Me-treated HCT116 cells **(N)**. GA-Me 1, GA-Me 2, and GA-Me 3 represent the three groups of HCT116 cells treated with GA-Me. Control 1, Control 2, and Control 3 represent three groups of untreated HCT116 cells.

Visualization of the expression ratios in a heatmap (**Figures 1A–D**), MA plot (**Figures 1E–H**), or volcano plot (**Figures 1I–L**) revealed the widespread extent of GA-Me-mediated regulation of the transcriptome. The heatmap indicated good clustering of samples. A clear distinction was observed between the treated and untreated groups, and the DElncRNA, DEcircRNA, DEmiRNA, and DEmRNA expression profiles were able to discriminate between the two groups (**Figures 1A–D**). **Figures 1E–H** shows the MA plots of the DElncRNA, DEcircRNA, DEmiRNA, and DEmRNA expression profiles between GA-Me-treated cells and untreated cells. The volcano plot shows a large number of statistically significant differences in the DElncRNA, DEcircRNA, DEmiRNA, and DEmRNA expression profiles (**Figures 1I–L**). Moreover, the ratio of differentially expressed RNAs (FC, illustrated in the abscissa of the volcano plot) was remarkable.

We performed a global overlapping gene analysis to further pinpoint the genes involved in the mechanism of GA-Me in CRC. Compared with the control group, 1572 DElncRNAs (807 downregulated and 765 upregulated), 123 DEcircRNAs (56 downregulated and 67 upregulated), 87 DEmiRNAs (58 downregulated and 29 upregulated), and 1508 DEmRNAs (911 downregulated and 597 upregulated) were observed after GA-Me treatment, and these altered RNAs are shown in **Figure 1M**. The intersections of DEmRNAs, DEmiRNA-target mRNAs, DElncRNA-target trans mRNAs, and DEcircRNA-host genes are shown in **Figure 1N** and **Table S5**. These results indicate two reliable core mRNAs that were validated by different platforms and across different institutions: discoidin, CUB and LCCL domain-containing protein 2 (DCBLD2) and Rap guanine nucleotide exchange factor 5 (RAPGEF5). DCBLD2 is a type-I transmembrane protein with significantly higher expression in CRC tissues than in adjacent normal tissues. High DCBLD2 expression was significantly associated with shorter overall survival (40), consistent with our findings that GA-Me significantly downregulated DCBLD2. RAPGEF5 expression is elevated in tumor samples compared to normal samples, indicating its involvement in tumorigenesis (41); in addition, the expression of the RAPGEF5 mRNA is upregulated after GA-Me treatment, indicating that it might be regulated at the protein level *via* posttranscriptional modification.

### GA-Me Inhibited the Proliferation of HCT116 Cells

**Figure 2:**
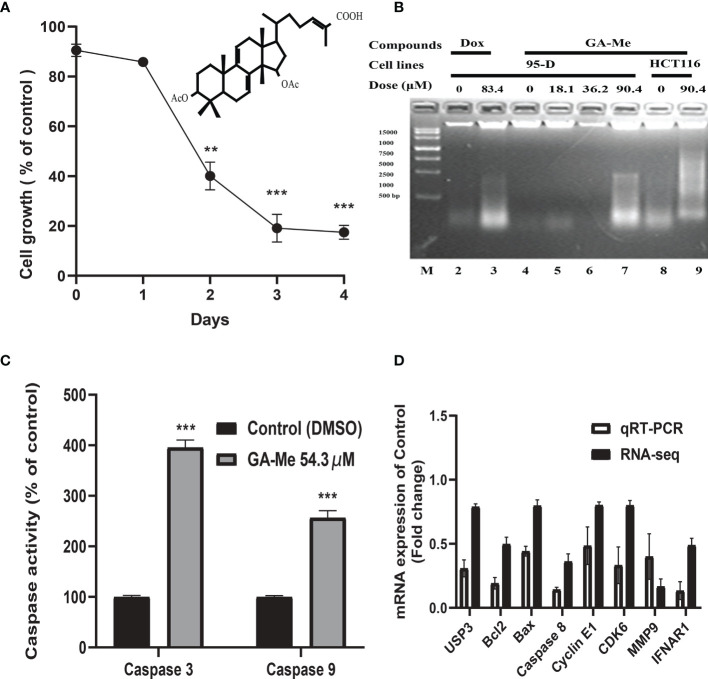
**(A)**: GA-Me inhibited the proliferation of HCT116 cells. After HCT116 cells were incubated with or without 18.1 µM GA-Me, the growth-inhibitory effects of GA-Me were compared by performing trypan blue staining and cell viability assays. Statistical significance of differences between DMSO and GA-Me groups: ** *p*<0.01 and *** *p*<0.001. **(B)**: GA-Me induced DNA fragmentation in HCT116 and 93-D cells. The indicated concentrations of GA-Me (*Lane 7*) and Dox (*Lane 3*, positive control) induced DNA fragmentation in 95-D cells; additionally, 90.4 µM GA-Me induced DNA fragmentation in HCT116 cells (*Lane 9*). The DNA marker is shown as *Lane M*. **(C)**: GA-Me significantly activates caspase-9 and caspase-3 in HCT116 cells. HCT116 cells were treated with or without 54.3 μM GA-Me for 24 hours. Caspase-3/7 and caspase-9 activity was measured using a Caspase-Glo-3/7 or Caspase-Glo-9 assay kit as described in “Materials and Methods”. Data are presented as the means ± SD. Statistical significance was determined using Student’s *t test*, where *** indicates *p* < 0.001 compared with 54.3 µM GA-Me. **(D)** The differential expression of mRNAs was validated using qRT–PCR. The expression levels of the UPS3, BCL2, BAX, caspase-8, Cyclin E1, CDK6, MMP9 and IFNAR1 mRNAs were downregulated in GA-Me-treated HCT116 cells compared to untreated HCT116 cells. The heights of the columns in the chart represent the FC. The qRT–PCR results were consistent with the RNA-Seq data. GA-Me represents HCT116 cells treated with GA-Me. Control represents untreated HCT116 cells.

In our previous study, GA-Me inhibited the growth of human colon carcinoma HCT116 cells (IC_50_ of 36.9 µM at 24 h) (16). We conducted a trypan blue staining cell viability assay to further study the effect of GA-Me on the proliferation of HCT116 cells. **Figure 2A** shows that 18.1 µM (approximately half of its IC_50,_ 10 µg/ml) GA-Me treatment obviously reduced the number of viable HCT116 cells on the indicated days. Thus, GA-Me inhibited the proliferation of HCT116 cells.

### GA-Me Induced DNA Fragmentation in HCT116 Cells

DNA fragmentation is a key feature of apoptosis. Many chemotherapeutic drugs that cause DNA damage are known to induce apoptosis (42). Here, the GA-Me-induced apoptotic response was confirmed by performing DNA fragmentation experiments. In **Figure 2B**, 83.4 μM Dox (positive control) and 90.4 μM GA-Me (approximately two-fold higher than its IC_50,_ 50 µg/ml) induced nuclear DNA fragmentation in 95-D cells (*Lanes 3 and 7*), whereas their controls (*Lanes 2* and *4*) did not. At the same time, GA-Me initiated DNA fragmentation in HCT116 cancer cells (**Figure 2B**, *Lanes 8 and 9*). The nuclear DNA of control cells was not obviously fragmented (*Lane 8*), and the HCT116 cells showed an apparent increase in nuclear DNA fragmentation when cultured in the presence of 90.4 μM GA-Me (*Lane 9*) for 24 h. These results confirmed that GA-Me induced DNA fragmentation in cancer cells.

### GA-Me Significantly Activates Caspase-9 and Caspase-3 in HCT116 Cells

We examined the effect of GA-Me on the downstream effectors of the mitochondrial cascade to analyze the downstream apoptotic pathway. As shown in **Figure 2C**, GA-Me treatment resulted in a significant increase in caspase-3 and caspase-9 activities, consistent with our previous result (16).

### Validation of the Expression Profile

Our previous and other studies reported that GA-Me regulated the apoptosis, cell cycle, invasion, immune and protein degradation pathways. Thus, we selected the UPS3, Bcl2, Bax, Caspase-8, Cyclin E1, CDK6, MMP9, and INFAR1 genes involved in these pathways to validate the accuracy and reliability of the RNA sequencing (RNA-Seq) results using qRT–PCR analysis. As shown in **Figure 2D**, the sequencing results were similar to the qRT–PCR results for the validated mRNAs.

### GO Classification and Enrichment Analysis

**Figure 3:**
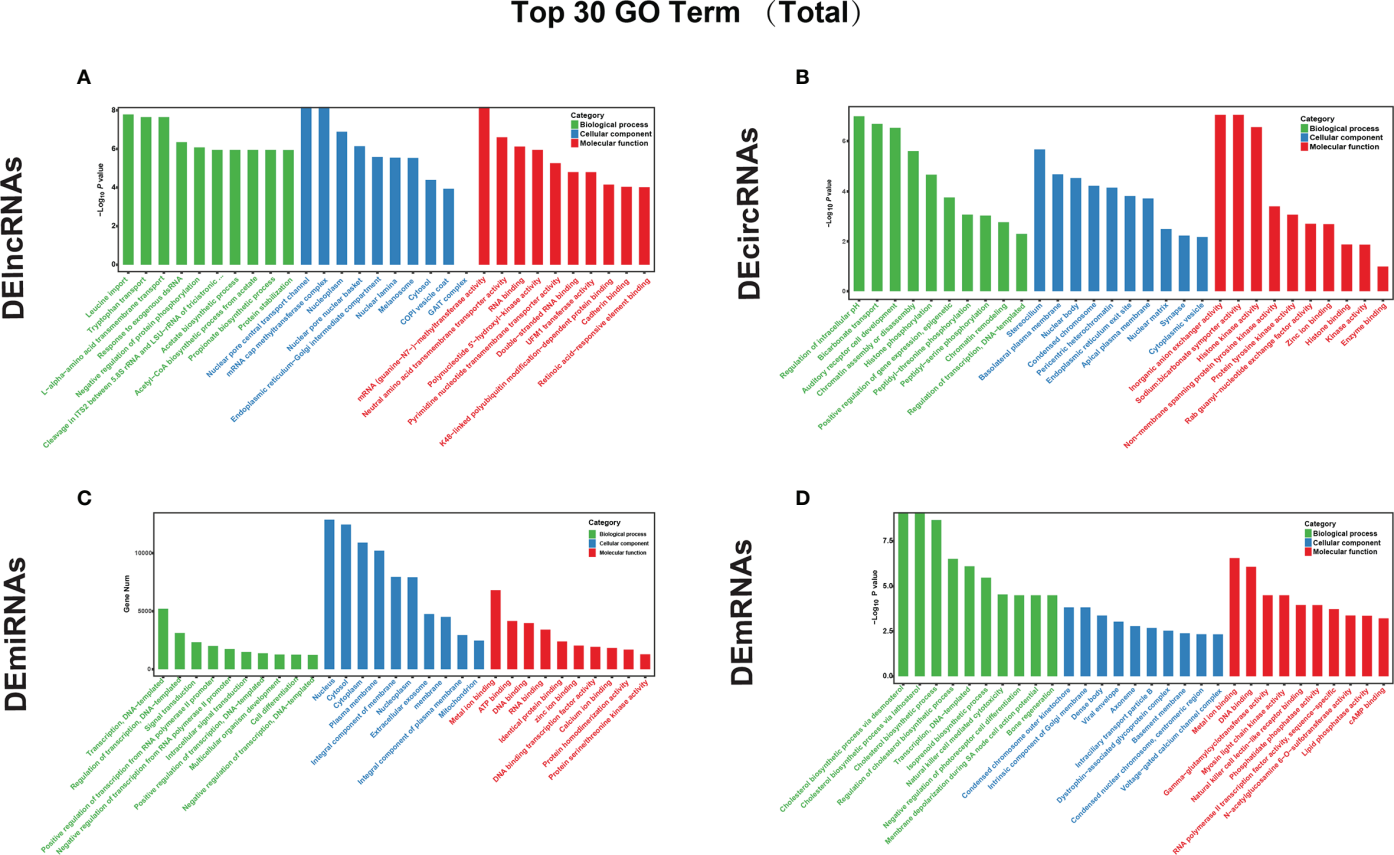
GO annotation was performed to analyze the functions of the top 30 DEGs. GO functional analyses of the top 30 **(A)** DElncRNAs, **(B)** DEcircRNAs, **(C)** DEmiRNAs, and DEmRNAs **(D)** between untreated and GA-Me-treated HCT116 cells. The x-axis represents the GO categories, and the y-axis represents the –log_10_ *^p^* ^value^ of the DEGs.

Based on the GO classification analysis of the total genes targeted by lncRNAs (**Figure 3A**), the most significantly enriched BP, CC, and MF terms were leucine import, negative regulation of protein phosphorylation, nuclear pore central transport channel, mRNA cap methyltransferase complex, mRNA (guanine-N7-)-methyltransferase activity, and polynucleotide 5’-hydroxyl-kinase activity. For the downregulated cis-targeted genes of lncRNAs (**Figure S1A**), the most significantly enriched BP, CC, and MF terms were midbrain morphogenesis, transcriptional repressor complex, and retinoic acid-responsive element, respectively. For the trans-targeted genes of lncRNAs (**Figure S1B**), the most significantly enriched BP, CC, and MF terms were negative regulation of protein autophosphorylation, mRNA cap methyltransferase complex, and mRNA (guanine-N7-)-methyltransferase activity, respectively.

Based on the GO classification of the total genes targeted by DEcircRNAs (**Figure 3B**), the most significantly enriched BP, CC, and MF terms were the regulation of intracellular pH, stereocilium, and inorganic anion exchanger activity, respectively. For the source genes of DEcircRNAs (**Figure S1C**), the most significantly enriched BP, CC, and MF terms among the downregulated factors were ER to Golgi vesicle-mediated transport, endoplasmic reticulum exit site, and chromatin binding, respectively. The most significantly enriched BP, CC, and MF terms among the upregulated factors were regulation of intracellular pH, stereocilium, and inorganic anion exchanger activity, respectively (**Figure S1D**).

Based on the GO classification of the total genes targeted by DEmiRNAs (**Figure 3C**), the most significantly top enriched BP, CC, and MF terms were regulation of transcription, DNA-templated; nucleus; and metal ion binding, respectively. For the downregulated target genes of DEmiRNAs (**Figures S1E, F**), the most significantly enriched BP, CC, and MF terms were regulation of photoreceptor cell differentiation, presynaptic membrane, and cAMP binding, respectively (**Figure S1E**). Conversely, the most significantly enriched BP, CC, and MF terms for the upregulated DEmiRNA target genes were regulation of bone regeneration, an intrinsic component of the Golgi membrane, and myosin light chain kinase activity, respectively (**Figure S1F**).

Based on the GO enrichment analysis of the total target genes of DEmRNAs (**Figure 3D**), the most significantly enriched BP, CC, and MF terms were the protein cholesterol biosynthetic process *via* desmosterol, condensed chromosome outer kinetochore, and metal ion binding, respectively. For the cis-targeted genes of mRNA (**Figure S1G**), the most significantly enriched BP, CC, and MF terms were protein transcription, DNA-templated; condensed chromosome outer kinetochore; and DNA binding, respectively. The most significantly enriched BP, CC, and MF terms for the trans-targeted genes of mRNAs (**Figure S1H**) were the stimulatory cholesterol biosynthetic process *via* desmosterol, dense bodies, and natural killer cell lectin-like receptor binding, respectively. The results of the GO analysis indicated that immune responses, lipid metabolism, infectious diseases, signal transduction, cell growth, and death pathways were activated in GA-Me-treated cells.

### KEGG Pathway Enrichment Analysis

**Figure 4:**
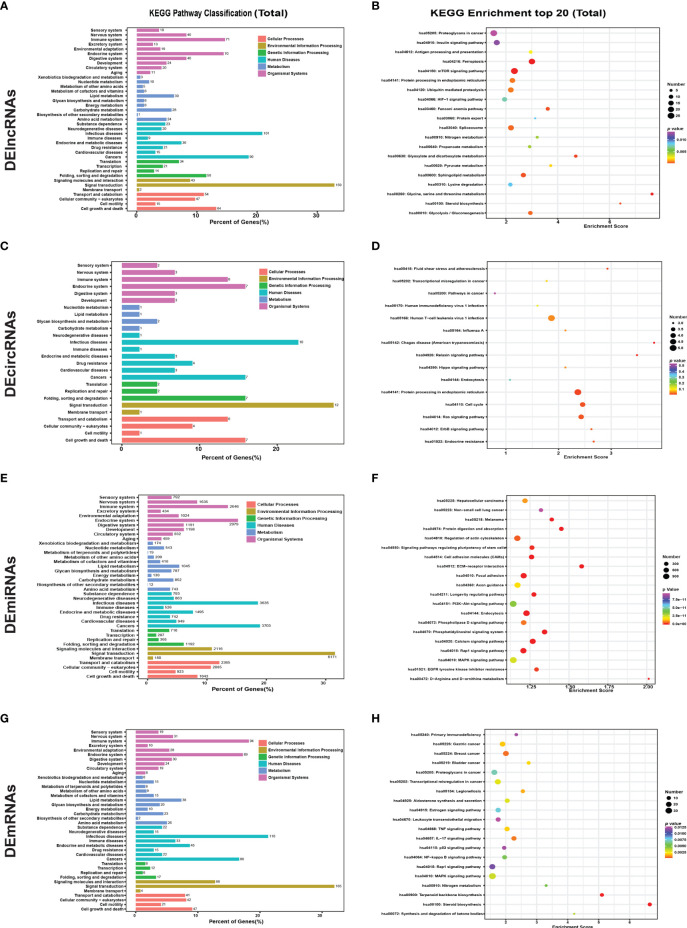
KEGG pathway analysis of DEGs in different groups and scatter plot of the top 20 enriched KEGG pathways. KEGG pathway enrichment analysis of all **(A)** DElncRNAs, **(C)** DEcircRNAs, **(E)** DEmiRNAs, and DEmRNAs **(G)** between untreated and GA-Me-treated HCT116 cells. Scatter plot of the top 20 enriched KEGG pathways. Top 20 KEGG pathways identified for all **(B)** DElncRNAs, **(D)** DEcircRNAs, **(F)** DEmiRNAs, and DEmRNAs **(H)** between untreated and GA-Me-treated HCT116 cells.

Gene set enrichment analysis of KEGG pathways was performed to clarify the biological processes in which the DEGs were involved, as shown in **Figures 4A–H** and **S2**; the DEGs were associated with pathways involved in cell growth and death, signal transduction, cancers, infectious diseases, lipid metabolism, and the endocrine system- and immune system-regulated gene expression of DElncRNA-targeted genes (**Figures S2A, B**), host genes of DEcircRNAs (**Figures S2C, D**), and targets of miRNAs (**Figures S2E, F**) and DEmRNAs (**Figures S2G, H**).

The most significantly enriched KEGG pathways are shown in **Figures 4** and **S3**. For the cis-targeted genes of DElncRNAs, ferroptosis and amino acid (glycine, serine, and threonine) metabolism were the most significantly enriched pathways (**Figure S3A**). For the trans-targeted genes of DElncRNAs (**Figure S3B**), steroid biosynthesis, pyrimidine metabolism, mTOR signaling pathway, and antigen processing and presentation were the most significantly enriched pathways. For the downregulated proteins corresponding to the host genes of DEcircRNAs, endocrine resistance and protein processing in the endoplasmic reticulum were the most significantly enriched pathways (**Figure S3C**); human T-cell leukemia virus 1 infection (**Figure S3D**) was the most significantly enriched pathway for the upregulated proteins. For the targets of miRNAs (**Figures S3E, F**), steroid biosynthesis, terpenoid backbone biosynthesis, and aldosterone synthesis and secretion were the most significantly enriched pathways. Nitrogen metabolism, RNA polymerase, and the IL-17 signaling pathway were the most significantly enriched pathways for the downregulated DEmRNAs (**Figure S3G**). Similarly, for the positively regulated proteins (**Figure S3H**), steroid biosynthesis and terpenoid backbone biosynthesis were the most significantly enriched pathways. The main biochemical pathways and signal transduction pathways determined by the KEGG analysis provide further insight into future research directions for ncRNAs and mRNAs. We further analyzed immune system-related genes, including MDM2, MMP2, MMP9, caspase-8, and other DEGs involved in the GA-Me-induced immune response after GA-Me treatment. These results also suggested that GA-Me is a multitarget ligand with polypharmacological efficacy in the treatment of CRC.

### ceRNA-Mediated Regulation of Interaction Networks

**Figure 5:**
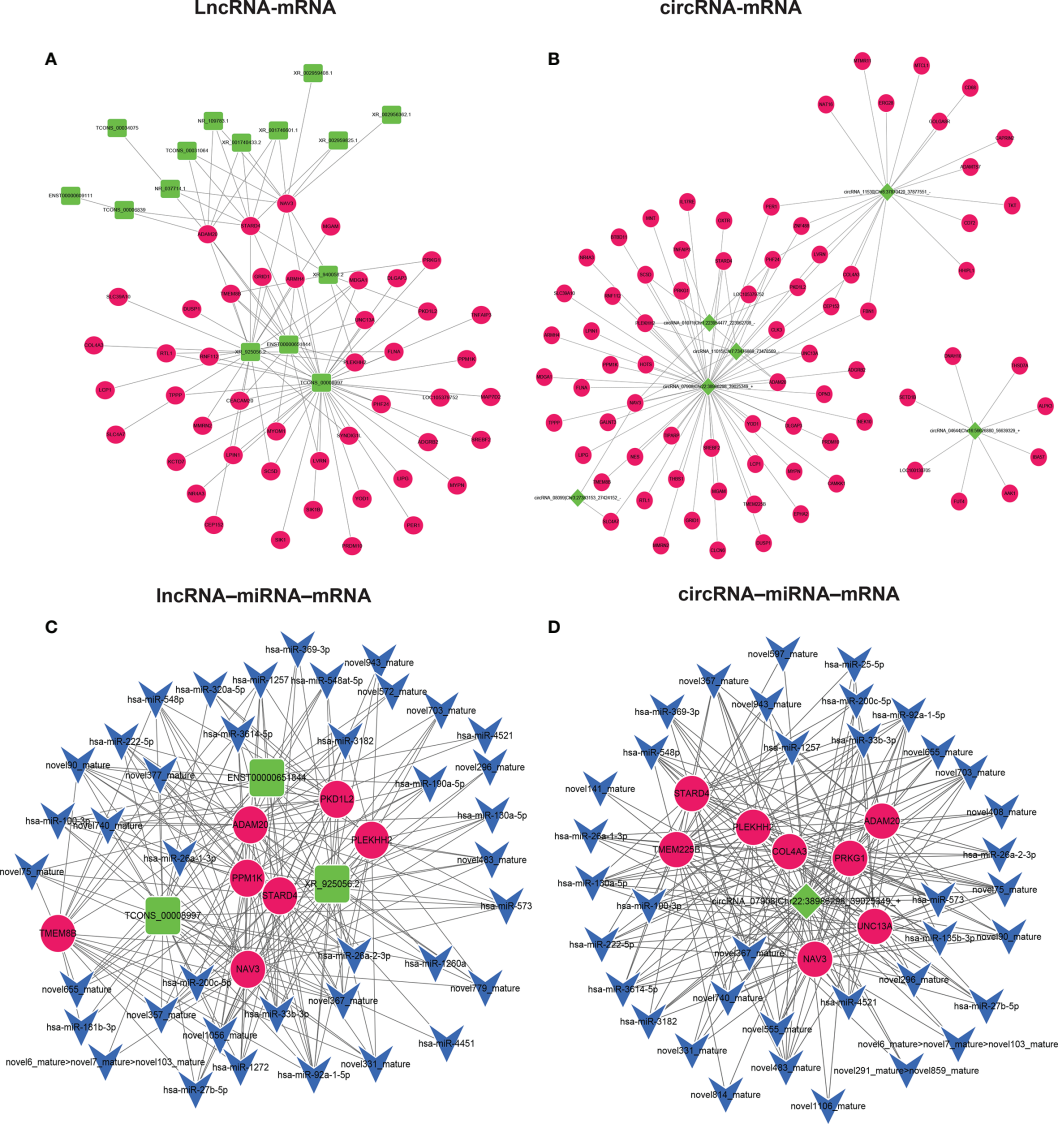
ceRNA–mRNA and ceRNA-miRNA–mRNA regulatory networks in GA-Me-treated HCT116 cells. **(A)** Interaction network of lncRNAs and mRNAs in HCT116 cells after GA-Me treatment. **(B)** Interaction network of circRNAs and mRNAs in HCT116 cells after GA-Me treatment. **(C)** Interaction network of lncRNAs, miRNAs, and mRNAs in HCT116 cells after GA-Me treatment. **(D)** Interaction network of circRNAs, miRNAs, and mRNAs in HCT116 cells after GA-Me treatment. Red and green represent up- and downregulation, respectively.

The study of the relationship between ncRNAs and mRNAs may increase our understanding of the molecular mechanisms of GA-Me against CRC. According to the ceRNA regulatory hypothesis, ncRNAs and mRNAs compete for the same miRNAs, resulting in additional layers of regulation of gene expression. Based on the analysis of DE lncRNAs, circRNAs, miRNAs, and mRNAs, a network of lncRNAs or circRNAs interacting with mRNAs was first constructed, as indicated in **Figures 5A, B**. **Figure 5A** illustrates our findings: 8 DElncRNAs interacted with 45 DEmRNAs. Interestingly, NR_109783.1, TCONS_00008997, TCONS_00031064, XR_001740433.2, XR_001746601.1, and XR_925056.2 regulated the ADAM20, STARD4, and NAV3 DEmRNAs. In addition, 6 DEcircRNAs interacted with 76 DEmRNAs after GA-Me treatment, as shown in **Figure 5B**. Importantly, circRNA-01071|Chr1:223954477-223962708, circRNA-07908|Chr22:38986298-39025349 and circRNA-11015|Chr7:73476868-73478569 regulated the ADAM20, STARD4, and NAV3 mRNAs after GA-Me treatment, which might be one of the reasons why GA-Me activated multiple targets to exert anti-CRC activity.

The circRNA–miRNA–mRNA regulatory network and lncRNA–miRNA–mRNA network were constructed based on the ceRNA theory to explore the molecular mechanisms of ncRNAs. Using lncRNAs as a decoy, miRNAs as the center, and mRNAs as a target, the lncRNA–miRNA–mRNA regulatory network that contained 7 mRNAs, 3 lncRNAs, and 39 miRNAs was built (**Figure 5C**). Using circRNAs as a decoy, miRNAs as the center, and mRNAs as the target, a circRNA–miRNA–mRNA regulatory network containing 8 mRNAs, 1 circRNA, and 37 miRNAs was generated (**Figure 5D**). In these two networks, different shapes represent different RNA types; red and green represent up- and downregulation, respectively. Interestingly, the circRNA-07908 (22:38986298|39025349)-hsa-miR-100-3p-NAV3, circRNA-07908 (22:38986298|39025349)-hsa-miR-100-3p-ADAM20, TCONS-00008997-hsa-miR-100-3p-NAV3 and TCONS-00008997-hsa-miR-100-3p-ADAM20 interactions implied that circRNA-07908 (22:38986298|39025349) regulated NAV3 and ADAM20 expression by competitively binding hsa-miR-100-3p. Other examples of this relationship are the TCONS-00008997-hsa-miR-1257-NAV3/ADAM20/STARD4, circRNA-07908|Chr22:38986298-39025349-hsa-miR-1257-NAV3/ADAM20/STARD4, XR-925056.2-hsa-miR-3182-NAV3/ADAM20/STARD4, circRNA-07908|Chr22:38986298-39025349-hsa-miR-3182-NAV3/ADAM20/STARD4, circRNA_07908|Chr22:38986298_39025349-hsa-miR-27b-5p-NAV3/STARD4, and TCONS_00008997-hsa-miR-27b-5p-NAV3/STARD4 interactions. These results suggest that circRNAs and lncRNAs harbor miRNA response elements and play pivotal regulatory roles in the polypharmacological mechanisms of GA-Me in CRC treatment.

**Figure 6:**
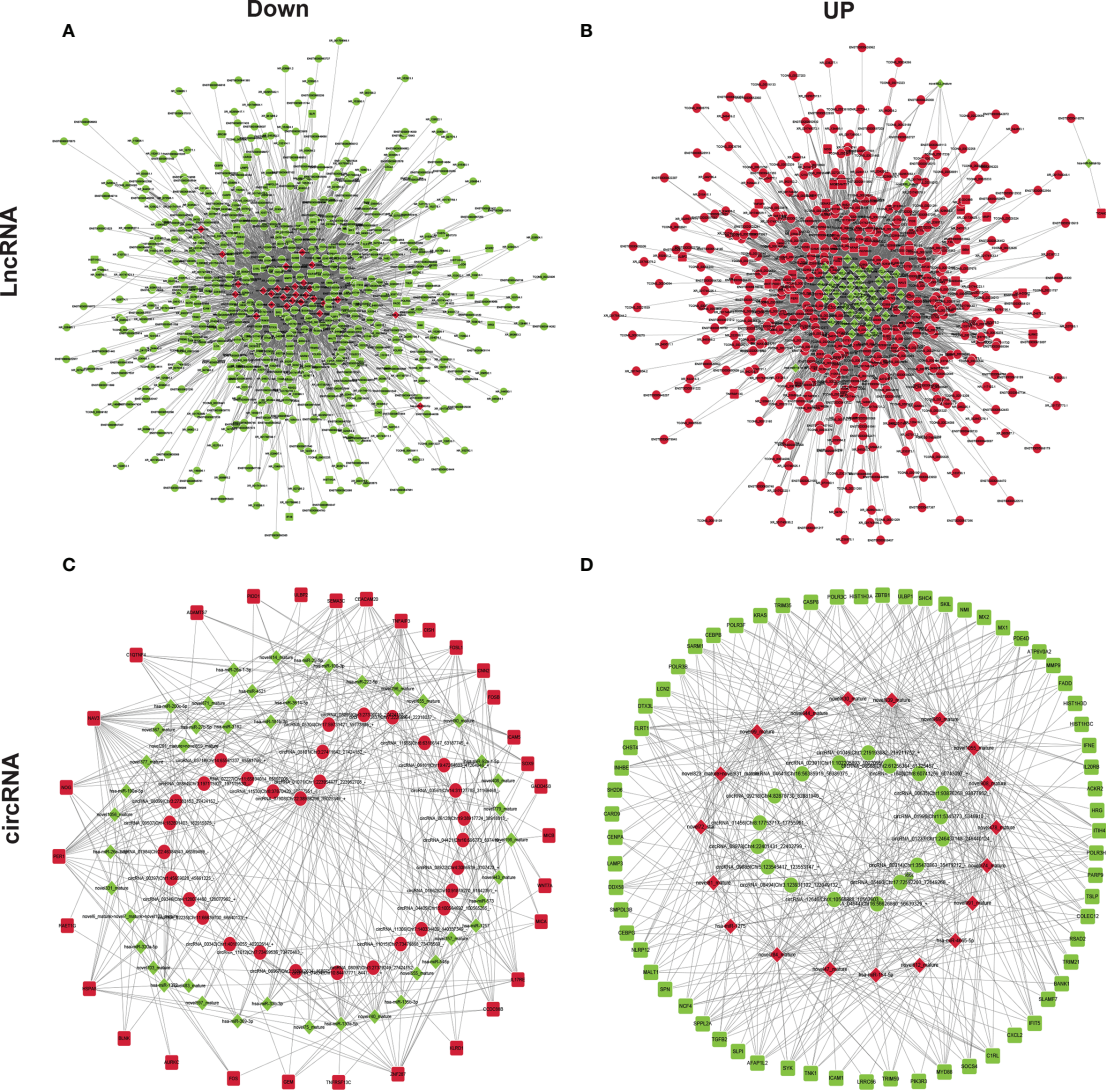
ceRNA–miRNA–mRNA regulatory immunological networks in GA-Me-treated HCT116 cells. **(A)** Downregulated components of the lncRNA–miRNA–mRNA interaction network in GA-Me-treated HCT116 cells. **(B)** Upregulated components of the lncRNA–miRNA–mRNA interaction network in GA-Me-treated HCT116 cells. **(C)** Downregulated components of the circRNA–miRNA–mRNA interaction network in GA-Me-treated HCT116 cells. **(D)** Upregulated components of the circRNA–miRNA–mRNA interaction network in GA-Me-treated HCT116 cells. Red and green represent up- and downregulation, respectively.

Previous studies reported that GA-Me clearly regulates immune function (12, 24). We next assessed the molecular mechanism by which ncRNAs modulate the immunological pathway after GA-Me treatment. The lncRNA–miRNA–mRNA regulatory immunological networks suggested that MMP9 was regulated by the novel874_mature miRNA, ENST00000414039 and ENST00000419190 after GA-Me treatment (**Figures 6A, B** and **Table S6**). The circRNA–miRNA–mRNA regulatory immunological networks suggested that MMP9 was downregulated by the novel874_mature miRNA, circRNA-00314|Chr1:35470863-35479212, and circRNA-05460|Chr17:72592203-72649268 after GA-Me treatment (**Figures 6C, D** and **Table S7**). The interactions between ncRNAs and the MMP9 mRNA suggested the activation of novel immunoregulatory mechanisms after GA-Me treatment, consistent with the results of the present study (**Figure 2D**) and our previous results (11). In addition, NAV3, STARD4, ADAM20 (**Figure 5**), and caspase-8 (**Figure 6**) participated in similar ceRNA regulatory ncRNA networks. These results also suggested that GA-Me might be a multitarget ligand with polypharmacological efficacy in terms of anti-CRC activity.

### Docking Analysis

**Figure 7:**
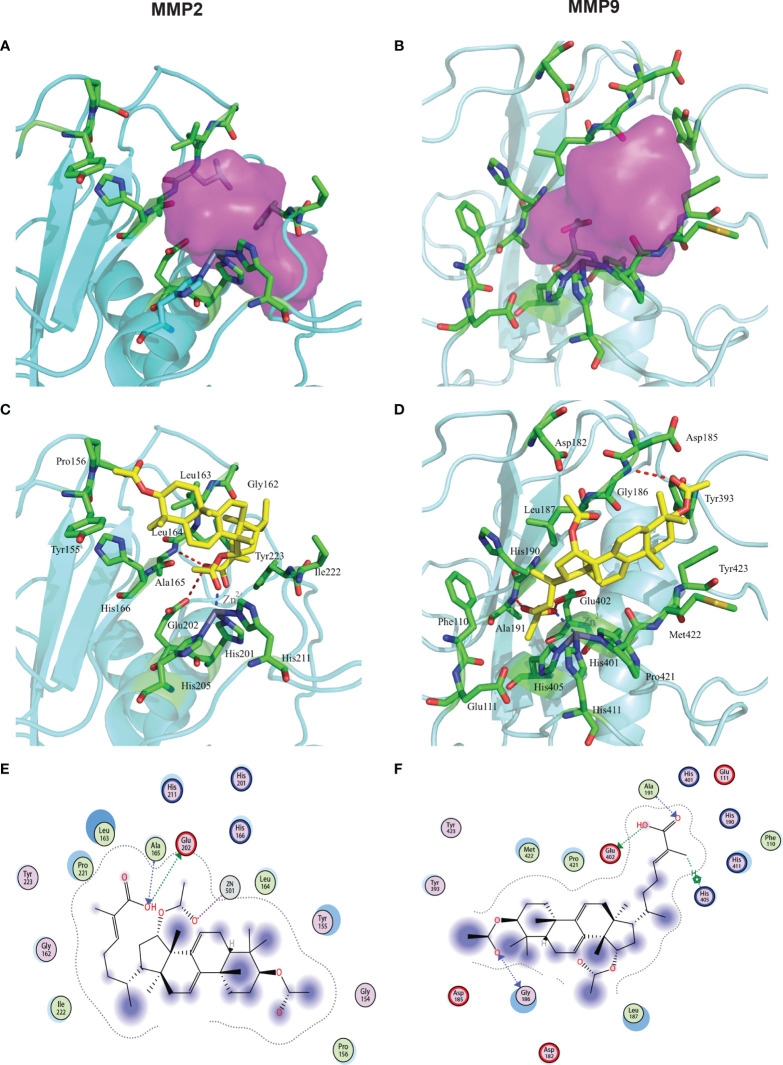
Interactions between MMP2/9 and GA-Me. The red dashed lines represent the hydrogen bonds between the ligands and targets. The blue dashed lines represent the metal contact. **(A)** Pocket information from the MMP2 crystal structure, highlighted in purple. **(B)** Pocket information from the MMP9 crystal structure, highlighted in purple. **(C)** The docking results for GA-Me and MMP2. The predicted *Kd* value of GA-Me is 7.02. **(D)** The docking results for GA-Me and MMP9. The predicted *Kd* value of GA-Me is 5.87. **(E)** 2D visualization of the interactions between GA-Me and MMP2. **(F)** 2D visualization of the interactions between GA-Me and MMP9. The important residues are circled.

In our previous studies, we showed that GA-Me decreased MMP2 and MMP9 mRNA and protein levels. These results also confirmed that GA-Me inhibited the expression of the MMP2 and MMP9 targets. We performed docking calculations *in silico* to further show that GA-Me also has the potential to bind to the ligand binding domain (LBD) of MMP2 and MMP9. We implemented SYBYL-X 1.3 in a molecular docking study to explore the detailed interactions between GA-Me and the MMP2 protein and the related molecular mechanisms, as shown in **Figure 7A**. The crystal structure of MMP2 (PDB entry: 1QIB) was downloaded from the PDB (34, 35). The MOLCAD module in SYBYL-X 1.3 was used to define the binding pocket of MMP2 (**Figure 7A**, purple area). Our predicted pocket was similar to the reported S1’ pocket of MMPs (36). Based on the pocket information, the detailed interactions between GA-Me and MMP2 were explored using the docking program Surflex-Dock GeomX (SFXC) in SYBYL-X 1.3. As shown in **Figure 7C**, hydrogen bonds and hydrophobic interactions coupled with conjugation to Zn^2+^ contributed to the high affinity of GA-Me for MMP2. Zn^2+^ is bound by three histidine residues (His201, His205, and His211) and forms a bond (2.0 Å) with the oxygen of GA-Me (**Figure 7E**). GA-Me may be a novel zinc-binding group (ZBG) MMP inhibitor. In addition to the interaction of GA-ME with Zn^2+^, the hydroxyl group of GA-Me donates a hydrogen, forming a hydrogen bond (3.2 Å) with the carboxyl group of Glu202, and accepts a hydrogen from the amino group of Ala165, forming another strong hydrogen bond (3.0 Å). Other residues (highlighted in green in **Figure 7**) within the binding pocket stabilize GA-Me by forming hydrophobic contacts. As reported in the literature, many of these highlighted residues are conserved in MMPs (34, 36). We obtained a docking score of 7.02 from the interactions between GA-Me and MMP2, indicating that we predicted a *Kd =* 10^-7.02^.

We used a similar approach to assess the interaction between GA-Me and MMP9. The cocrystal structure of the MMP9 complex with a reverse hydroxamate inhibitor (PDB entry: 1GKC) was acquired from the PDB (37). The original ligand, STN-BUM, was used to predict the binding pocket. Afterward, it was removed in PyMOL to avoid unnecessary blocks and interactions (38). The docking results showed several hydrogen bonds, hydrophobic contacts, and conjugation to Zn^2+^-stabilized GA-Me within a defined pocket. As shown in **Figure 7B**, the amino group of Ala191 donates a hydrogen to the carboxyl group of GA-Me (2.9 Å). In addition, the nearby carboxyl group of Glu402 accepts a hydrogen from the hydroxyl group of GA-Me (3.0 Å). The oxygen of GA-Me also forms a bond (2.6 Å) with Zn^2+^ (**Figure 7D**). These three close interactions coupled with hydrophobic contacts with the surrounding nonpolar residues contribute to the stabilization of the carboxyl moiety of GA-Me within the part of the pocket near the Zn^2+^ bond. The other side of GA-Me interacts with MMP9 by forming a hydrogen bond with Gly186 (3.3 Å). The four-membered ring moiety of GA-Me is fixed within the pocket by forming hydrophobic contacts with the surrounding residues (**Figure 7F**). The total score of GA-Me binding to MMP9 was 5.87, with a predicted *Kd* = 10^-5.87^. Therefore, the docking results showed that GA-Me binds to the LBDs of human MMP2/9 and suggested that GA-Me might be an MMP2 and MMP9 inhibitor. These results matched our experimental results (**Figure 2**) and previous results (11). According to our predictions from the molecular docking study, mutation of the key residues listed above are worth performing in the future.

### PPI Network

**Figure 8:**
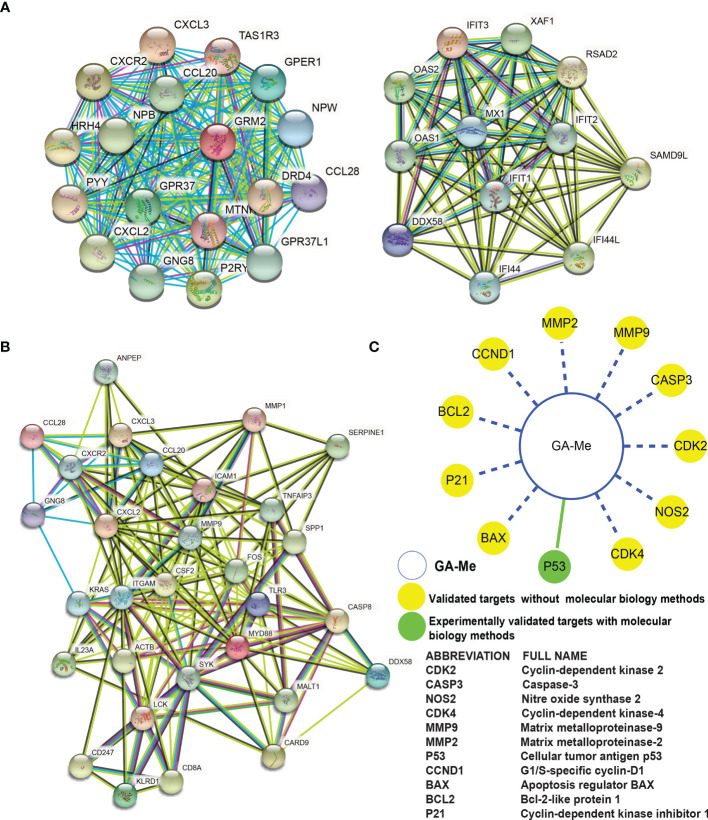
PPI network analysis of DEGs in GA-Me-treated and untreated HCT116 cells. **(A)** The PPI network showed the top 30 hub DEGs among the total DEGs and their interactions with each other. **(B)** The PPI network showed the top 30 hub DEGs among 94 immune-related DEGs and their interactions with each other. The darker the color, the more critical the effect. Line colors indicate the type of interaction evidence (red: predicted interaction based on gene fusion; yellow: text mining; green: predicted interaction based on gene neighborhood; blue: predicted interaction based on gene cooccurrence; light blue: known interaction from a curated database; purple: known interaction experimentally determined; black: coexpression; lilac: protein homology). (C) Systems pharmacological analysis of GA-Me. The green solid line indicates experimentally verified ligand-target interactions. The blue dashed line indicates predicted ligand-target interactions.

From the 1508 identified DEmRNAs identified from GA-Me-treated versus untreated HCT116 cell samples, 1443 nodes and 5461 interacting edges were obtained for the PPI network analysis using the STRING database. As shown in **Figure 8A**, a topological analysis was performed using CytoHubba, a Java plug-in for Cytoscape software, to analyze the 1443 nodes and identified 30 key nodes: GNG8, CXCL2, CCL20, CXCR2, CXCL3, DRD4, GRM2, PYY, GPER1, CCL28, GPR37, TAS1R3, NPB, P2RY4, HRH4, MTNR1A, NPW, GPR37L1, MX1, IFIT1, OAS1, RSAD2, OAS2, IFIT3, IFIT2, IFI44, IFI44L, XAF1, SAMD9L, and DDX58. These nodes were considered key proteins in the whole network and ultimately constituted 219 key interactions (**Figure 8A**). The CXCR2 protein was the key node with the highest degree in the GA-Me response network.

Among the top 94 DEGs involved in the immune response after GA-Me treatment, the topological PPI network analysis using the STRING database revealed 30 nodes, namely, CSF2, TLR3, ICAM1, MMP9, ITGAM, CXCL2, MYD88, CCL20, CXCR2, FOS, CASP8, SYK, ACTB, KRAS, IL23A, SPP1, MMP1, CXCL3, SERPINE1, LCK, KLRD1, CD8A, TNFAIP3, CARD9, DDX58, MALT1, ANPEP, GNG8, CCL28, and CD247, and identified 170 key interacting edges (**Figure 8B**). The MMP9 and CASP8 proteins were the key nodes involved in the immune response during GA-Me treatment of CRC cells.

Overall, the PPI network analyses suggest that high-degree nodes (GNG8, CXCL2, CCL20, CXCR2, CXCL3, CCL28, and DDX58) are clustered in the immune response.

### Polypharmacological Analysis of GA-Me

In **Figure 8C**, the green solid line indicates experimentally verified ligand-target interactions. The blue dashed line indicates predicted ligand-target interactions. Notably, p53 was experimentally validated as a target in our previous paper, and ten other targets were predicted using the CRC HTDocking platform (http://www.cbligand.org/CRC/docking_search.php) and data from previous reports (43).

All results strongly suggested that GA-Me is a multitarget ligand with polypharmacological efficacy in CRC treatment.

## Discussion

Multiple studies have documented the anti-CRC efficacy of GA-Me (11, 12, 16, 17, 24, 25), but the exact mechanism underlying its antitumor effects remains unclear. To the best of our knowledge, this comprehensive report of lncRNAs, circRNAs, miRNAs, and mRNAs reveals regulatory pathways involved in the anti-CRC efficacy of GA-Me. In general, our previous data confirmed that GA-Me inhibits proliferation and induce apoptosis in HCT116 cells. With FC ≥ 2.0 and *p* value < 0.05 as the thresholds, 1572 lncRNAs, 123 circRNAs, 87 miRNAs, and 1508 mRNAs with significant differential expression were identified in GA-Me-treated cells compared with untreated cells (**Figure 1A**). We found that several DEmRNAs and DEmiRNAs may be associated with the anti-CRC efficacy of GA-Me. However, a large majority of the DElncRNAs and DEcircRNAs had not been previously identified, mainly due to the lack of research in this area. Moreover, 8 identified dysregulated mRNAs were selected for qRT–PCR validation, and the results confirmed the sequencing findings to some extent (**Figure 2**). Based on the KEGG analysis, all four RNAs were significantly enriched in three pathways: MAPK signaling, IL-17 signaling, and the p53 signaling pathway. Previous studies have reported that the p53 signaling pathway may be the possible molecular mechanism of GA-Me treatment of CRC (16). Our research results confirmed this hypothesis in a more comprehensive and systematic manner.

First, we focused on the differentially expressed coding genes. The MYC, KRAS, TGFB2, PIK3R3, CDKN1A, FOS, and GADD45B DEmRNAs were regarded as the most important DEmRNAs involved in the mechanism by which GA-Me treats CRC, as their dysregulation might result in the progression of CRC. The KEGG pathway analysis indicated that GA-Me-responsive gene alterations in HCT116 cells were significantly enriched in the IL-17 signaling pathway, a widely known immune pathway. MMP9 and caspase-8 were enriched in the IL-17 signaling pathway, which has been strongly associated with shorter overall survival of patients with CRC (44). MMP9 was consistently upregulated in primary colorectal tumors (45, 46). In the present study, MMP9 was proven to be downregulated in GA-Me-treated cancer cells compared to untreated cancer cells, consistent with our previous study (11). These investigations indicated that MMP9 might be one of the potential anti-CRC targets of GA-Me that facilitates the regulation of the IL-17 signaling pathway.

MMP9 was regulated by the novel874_mature miRNA and ENST00000414039 and ENST00000419190 after GA-Me treatment (**Figures 6A, B**). The circRNA–miRNA–mRNA regulatory immunological networks suggested that MMP9 was regulated by the novel874_mature miRNA, circRNA-00314|Chr1:35470863-35479212, and circRNA-05460|Chr17:72592203-72649268 after GA-Me treatment (**Figures 6C, D**). The interactions between ncRNAs and the MMP9 mRNA suggest the activation of novel immunoregulatory mechanisms by GA-Me treatment.

The effects of GA-Me on ncRNAs, including miRNAs, lncRNAs, and circRNAs, were also evaluated in the current study. Previous studies indicated that hsa-miR-3182 (47) and hsa-miR-27b-5p (48) dysregulation may contribute to the progression of CRC. In our study, hsa-miR-27b-5p and hsa-miR-3182 were significantly downregulated in GA-Me-treated HCT116 cells compared to untreated cells. Thus, hsa-miR-27b-5p and hsa-miR-3182 may be important in the anti-CRC mechanism of GA-Me. Additionally, four DEmiRNAs were identified as the most likely candidate miRNAs associated with the mechanism of GA-Me. Among these, hsa-miR-100-3p and hsa-miR-1257 were expressed at significantly lower levels in CRC tissues than in normal tissues. In our research, these two miRNAs were downregulated in GA-Me-treated HCT116 cells compared to control cells. These observations suggest that hsa-miR-100-3p and hsa-miR-1257 may play vital roles in the anti-CRC mechanism of GA-Me. Furthermore, some DEmiRNAs, such as novel1056_mature, novel1056_mature, novel296_mature, novel331_mature, novel357_mature, novel377_mature, novel483_mature, novel572_mature,novel655_mature,novel703_mature, novel740_mature, novel75_mature, novel779_mature, novel90_mature, and novel943_mature, were also significantly differentially expressed between CRC cells treated with and without GA-Me, suggesting that these miRNAs play important roles in the anti-CRC mechanism of GA-Me.

We noticed that protein degradation was a significantly enriched GO term among DElncRNAs and their target genes. This phenomenon is very illuminating, given the importance of protein stabilization and K48-linked polyubiquitin modification-dependent protein binding in cancer (**Figure 3A**). Consistent with the results from the GO analysis, the KEGG pathway analysis also revealed that protein folding, sorting, and degradation (**Figure 4A**) and ubiquitin-mediated proteolysis (**Figure 4B**) were among the top enriched pathways.

The lncRNA–miRNA–mRNA network analysis revealed that TCONS_00008997 and XR-925056.2 were coexpressed with NAV3, which plays important roles in the anti-CRC mechanisms of GA-Me through competitively binding to hsa-miR-3182. Additionally, ENST00000651844 was identified to competitively bind hsa-miR-3182 and subsequently regulate ADAM20 expression. These lncRNAs were first reported to have functions in the effect of GA-Me on CRC.

Based on accumulating evidence, circRNAs modulate miRNA activity by functioning as endogenous sponges and affect mRNA splicing and transcription by interacting with the Pol II complex in the nucleus. As many circRNAs are not allocated to functional modules, limited public data about these circRNAs are available. In this study, based on the constructed circRNA–miRNA–mRNA coexpression network, we observed that one important circRNA contained one or more miRNA binding sites. Thus, circRNA_07908|Chr22:38986298_39025349 interacts with NAV3 through competitive binding with hsa-miR-3182. This competitive binding mode was similar to that by which hsa-miR-100-3p, hsa-miR-1257, and hsa-miR-27b-5p regulate NAV3. Therefore, further study is warranted to reveal the interaction relationships of circRNA_07908|Chr22:38986298_39025349–hsa-miR-3182–NAV3 in the mechanism of action of GA-Me. Although altered ncRNAs and mRNAs were identified and their possible roles in the anti-CRC mechanisms of GA-Me were investigated, several limitations should be considered when interpreting our findings. According to these results, circRNAs and lncRNAs harbor miRNA response elements and play pivotal regulatory roles in the polypharmacological mechanisms of GA-Me in CRC treatment.

Our previous studies reported that GA-Me targets and affect MMP2, MMP9 (11), IL-2, IFN-γ (12), caspase-9, caspase-3, STAT1, JAK1 (24), MDR1, MRP1, MRP2 (25), Bax, Bcl-2, Cyto-c (16, 25), and p53 (16, 17, 25) expression in GA-Me-treated cancer cells, implying that GA-Me might be a multitarget ligand with polypharmacological efficacy in CRC treatment (**Figure 8C**). However, those nonsystematic studies did not show the whole profile and network underlying the effects of GA-Me on cancers. Additionally, these analyses were only performed on HCT116 cells. Global changes in ncRNA and mRNA expression require further study to more accurately elucidate the anti-CRC mechanisms of GA-Me in other CRC cell lines and CRC animal models. One limitation of RNA-Seq technology is that inaccessible transcriptome complexities are difficult to determine. Importantly, the functions of ncRNAs remain mostly unknown, and the interpretation of our data is not straightforward. Further studies should include a proteomic analysis of expression profiles in GA-Me-treated and untreated cells to solve these problems. Finally, future studies should include knockout or overexpression of the TCONS_00008997, XR-925056.2 circRNA_07908, and hsa-miR-3182 ncRNAs and the NAV3 and DCBLD2 mRNAs to validate these results using molecular methods and to further confirm the main targets of GA-Me in CRC treatment. Overall, the PPI network analyses suggested that the high-degree nodes (GNG8, CXCL2, CCL20, CXCR2, CXCL3, CCL28, and DDX58) were clustered in the immune response. Additional experiments that manipulate the expression of the top potential targets identified in this study are needed to further elucidate the mechanisms by which GA-Me treats CRC.

## Conclusions

Drug discovery usually focuses on highly selective drugs to avoid potential side effects. However, drug research has recently tended toward systems-level polypharmacology, a more systems biology-oriented approach that considers the pleiotropy of biological networks at the molecular and cellular levels (26); this approach facilitates the identification of ligands that hit a set of selected, therapeutically relevant targets. Small-molecule compounds interact with diverse targets individually or simultaneously (27).

Whole-transcriptome sequencing and bioinformatics analysis of HCT116 cells treated with and without GA-Me were performed, and these results might provide a better understanding of the potential roles of lncRNAs, circRNAs, miRNAs, and mRNAs in the anti-CRC efficacy of GA-Me. This study suggested that GA-Me is a potential multitarget lead compound for CRC treatment with unique polypharmacological advantages, corresponding roles and molecular mechanisms, especially the polypharmacological mechanisms of these ncRNAs and mRNAs. This study provides a useful example of the study of the multitarget mechanism of GA-Me, in particular, the study of western medicine of this traditional Chinese medicine. This research has also laid a certain theoretical foundation for the in-depth development of traditional Chinese herbal medicine, which provides insights for follow-up studies and should be further explored in the future, such as we would investigate the effect of GA-Me on additional CRC cells to further focus on the broad spectrum antitumor effect and mechanism in CRC cells, perform whole-transcriptome profiling and bioinformatics analysis of paired CRC and adjacent normal tissue after GA-Me treatment in the CRC HCT116 tumor xenograft mouse model, including the unique DElncRNAs, DEcircRNAs, DEmRNAs and DEmiRNAs regulated by GA-Me in the future.

## Data Availability Statement

The original contributions presented in the study are included in the article/**Supplementary Material**. Further inquiries can be directed to the corresponding authors.

## Author Contributions

NC contributed to the conception of the design, conducted the experiments and data analysis, and drafted and revised the final version of the manuscript. GW conducted the qPCR experiments. XZ provided some important suggestions and made critical revisions. All authors have reviewed the final version of the manuscript and approved the final manuscript for publication.

## Funding

This work was supported by grants from the Shanghai Science and Technology Commission (No. 054319933) and the Shanghai Municipal Education Commission-Young Teacher Training Projection Program (ZZJKYX18004).

## Conflict of Interest

The authors declare that the research was conducted in the absence of any commercial or financial relationships that could be construed as a potential conflict of interest.

## Publisher’s Note

All claims expressed in this article are solely those of the authors and do not necessarily represent those of their affiliated organizations, or those of the publisher, the editors and the reviewers. Any product that may be evaluated in this article, or claim that may be made by its manufacturer, is not guaranteed or endorsed by the publisher.

## Supplementary Material

Supplementary Figure 1 Top 30 significantly enriched up- and downregulated GO terms in GA-Me-treated HCT116 cells. GO enrichment analysis of the cis-targeted genes of DElncRNAs **(A)**, the trans-targeted genes of DElncRNAs **(B)**, the host genes that were downregulated **(C)** and upregulated by DEcircRNA targeting **(D)**, and the genes that were downregulated **(E)** and **(F)** by upregulated DEmiRNA targeting. The downregulated **(G)** and upregulated **(H)** DEmRNAs identified after GA-Me treatment are shown. The GO enrichment analysis provided a controlled vocabulary for describing the coexpressed genes of the DEncRNAs and DEmRNAs. The GO terms covered three domains: BP, CC, and MF. The abscissa represents the number of genes annotated in the GO term, the ordinate represents the GO term, and the color of the column represents the corrected p value. GO, Gene Ontology.

Supplementary Figure 2 KEGG pathway analysis of DEGs in different groups of GA-Me-treated HCT116 cells. KEGG pathway enrichment analysis of the cis-targeted genes of DElncRNAs **(A)**, the trans-targeted genes of DElncRNAs **(B)**, the host genes that were downregulated **(C)** and upregulated by DEcircRNA targeting **(D)**, and the genes that were downregulated **(E)** and upregulated **(F)** by DEmiRNA targeting. The downregulated **(G)** and upregulated **(H)** DEmRNAs identified after GA-Me treatment are shown. The abscissa gene ratio represents the proportion of genes of interest in the pathway, and the ordinate represents each pathway. The size of the dots represents the number of genes annotated in the pathway, and the color of the dots represents the corrected *p* value from the hypergeometric test. KEGG, Kyoto Encyclopedia of Genes and Genomes.

Supplementary Figure 3 Scatter plot of the top 20 enriched KEGG pathways in GA-Me-treated HCT116 cells. The top 20 KEGG pathways enriched among the cis-targeted genes of DElncRNAs **(A)**, the trans-targeted genes of DElncRNAs **(B)**, the host genes that were downregulated **(C)** and upregulated by DEcirRNA targeting **(D)**, and the genes that were downregulated **(E)** and upregulated **(F)** by DEmiRNA targeting. The downregulated **(G)** and upregulated **(H)** DEmRNAs identified after GA-Me treatment are shown. The abscissa enrichment score represents the proportion of genes of interest in the pathway, and the ordinate represents each pathway. The size of the dots represents the number of genes annotated in the pathway, and the color of the dots represents the corrected *p* value of the hypergeometric test. KEGG, Kyoto Encyclopedia of Genes and Genomes.

## Abbreviations

GA-Me, ganoderic acid Me; GA-T, ganoderic acid T; DEGs, differentially expressed genes; GO, Gene Ontology; KEGG, Kyoto Encyclopedia of Genes and Genomes; BP, biological process; MF, molecular function; CC, cellular component; DMSO, dimethyl sulfoxide; PBS, phosphate-buffered saline; qRT–PCR, quantitative real-time polymerase chain reaction; DElncRNAs, differentially expressed long noncoding RNAs; DEcircRNAs, differentially expressed circular RNAs; DEmiRNAs, differentially expressed microRNAs; DEmRNAs, differentially expressed messenger RNAs; MMPs, matrix metalloproteinases.
