# Supplementary figures and images for: Integrated Whole-Transcriptome Profiling and Bioinformatics Analysis of the Polypharmacological Effects of Ganoderic Acid Me in Colorectal Cancer Treatment
Source: Front Oncol. 2022 Apr 27;12:833375. doi: 10.3389/fonc.2022.833375 (PMC9093067; doi:10.3389/fonc.2022.833375)

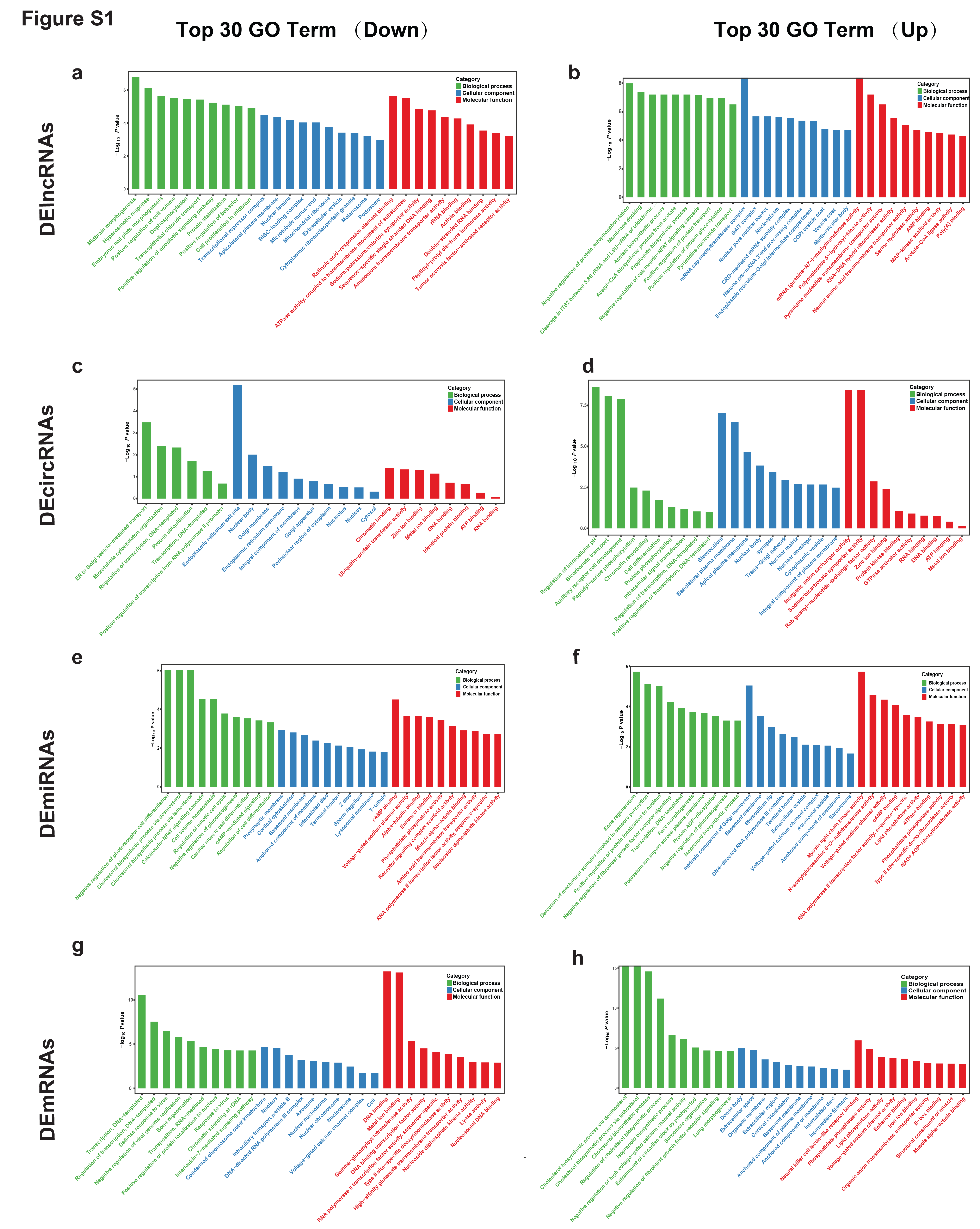

Supplement: Supplementary Figure 1 — Top 30 significantly enriched up- and downregulated GO terms in GA-Me-treated HCT116 cells. GO enrichment analysis of the cis-targeted genes of DElncRNAs (A), the trans-targeted genes of DElncRNAs (B), the host genes that were downregulated (C) and upregulated by DEcircRNA targeting (D), and the genes that were downregulated (E) and (F) by upregulated DEmiRNA targeting. The downregulated (G) and upregulated (H) DEmRNAs identified after GA-Me treatment are shown. The GO enrichment analysis provided a controlled vocabulary for describing the coexpressed genes of the DEncRNAs and DEmRNAs. The GO terms covered three domains: BP, CC, and MF. The abscissa represents the number of genes annotated in the GO term, the ordinate represents the GO term, and the color of the column represents the corrected p value. GO, Gene Ontology. [file Image_1.tif]

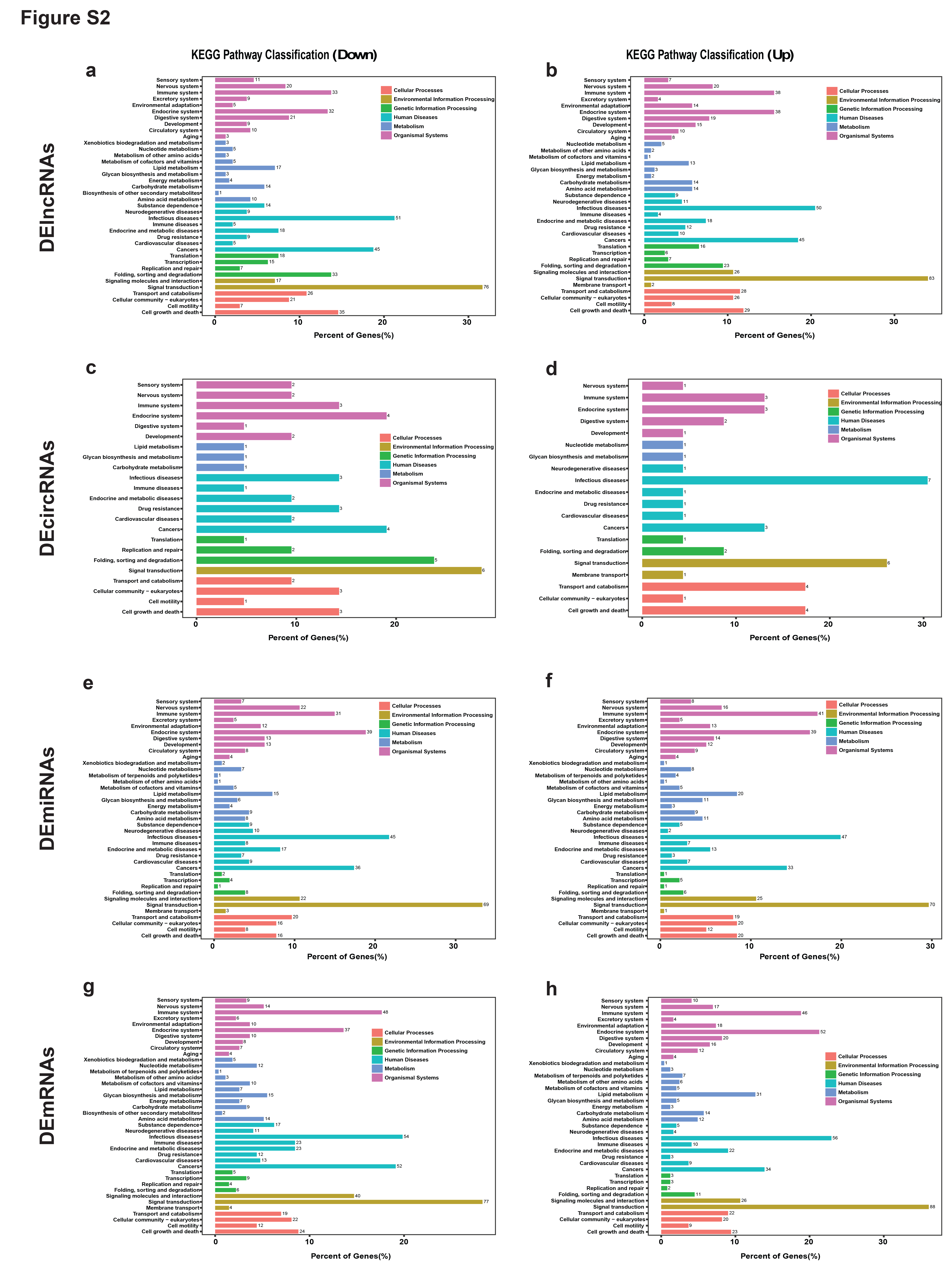

Supplement: Supplementary Figure 2 — KEGG pathway analysis of DEGs in different groups of GA-Me-treated HCT116 cells. KEGG pathway enrichment analysis of the cis-targeted genes of DElncRNAs (A), the trans-targeted genes of DElncRNAs (B), the host genes that were downregulated (C) and upregulated by DEcircRNA targeting (D), and the genes that were downregulated (E) and upregulated (F) by DEmiRNA targeting. The downregulated (G) and upregulated (H) DEmRNAs identified after GA-Me treatment are shown. The abscissa gene ratio represents the proportion of genes of interest in the pathway, and the ordinate represents each pathway. The size of the dots represents the number of genes annotated in the pathway, and the color of the dots represents the corrected p value from the hypergeometric test. KEGG, Kyoto Encyclopedia of Genes and Genomes. [file Image_2.tif]

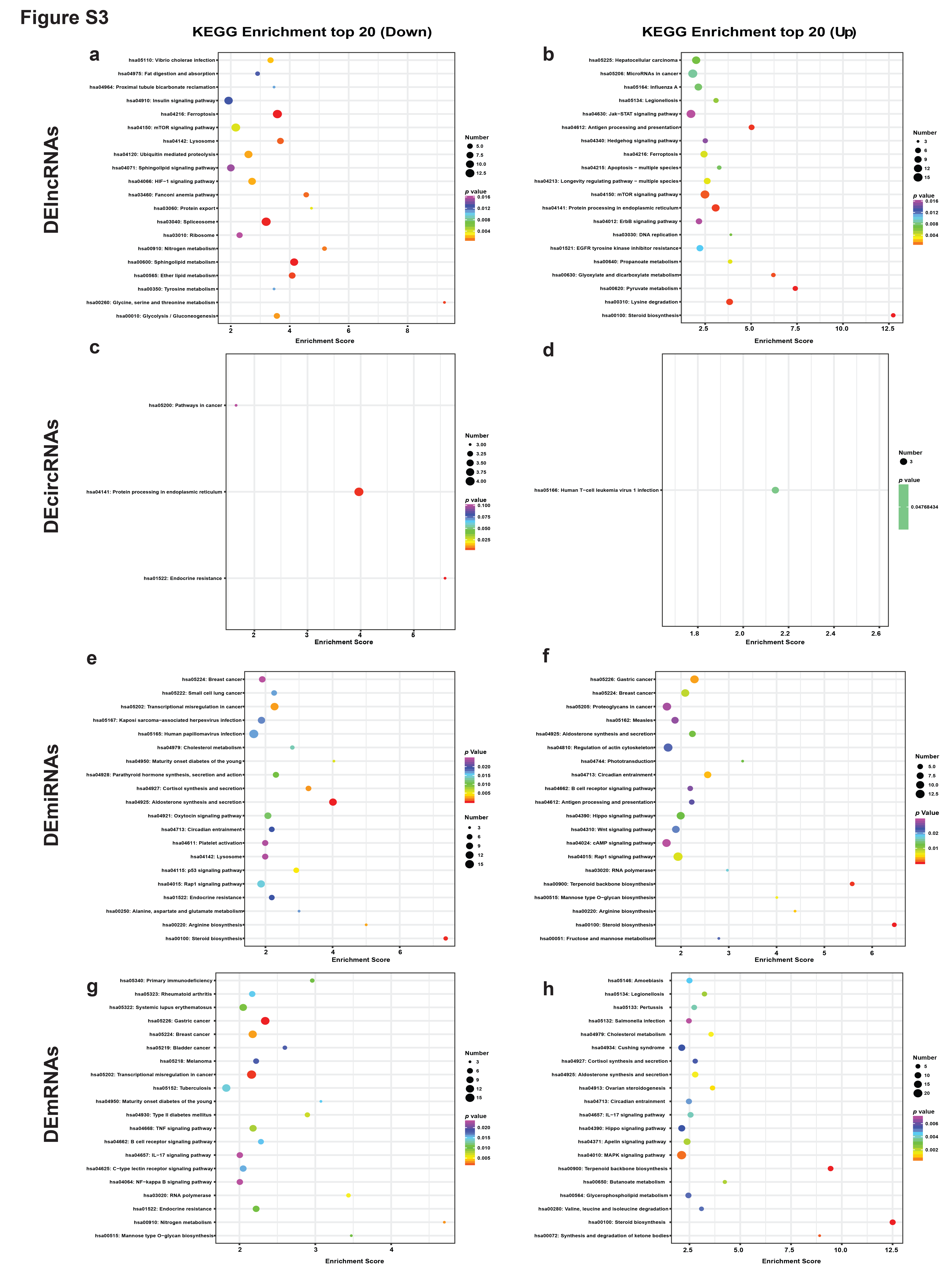

Supplement: Supplementary Figure 3 — Scatter plot of the top 20 enriched KEGG pathways in GA-Me-treated HCT116 cells. The top 20 KEGG pathways enriched among the cis-targeted genes of DElncRNAs (A), the trans-targeted genes of DElncRNAs (B), the host genes that were downregulated (C) and upregulated by DEcirRNA targeting (D), and the genes that were downregulated (E) and upregulated (F) by DEmiRNA targeting. The downregulated (G) and upregulated (H) DEmRNAs identified after GA-Me treatment are shown. The abscissa enrichment score represents the proportion of genes of interest in the pathway, and the ordinate represents each pathway. The size of the dots represents the number of genes annotated in the pathway, and the color of the dots represents the corrected p value of the hypergeometric test. KEGG, Kyoto Encyclopedia of Genes and Genomes. [file Image_3.tif]
